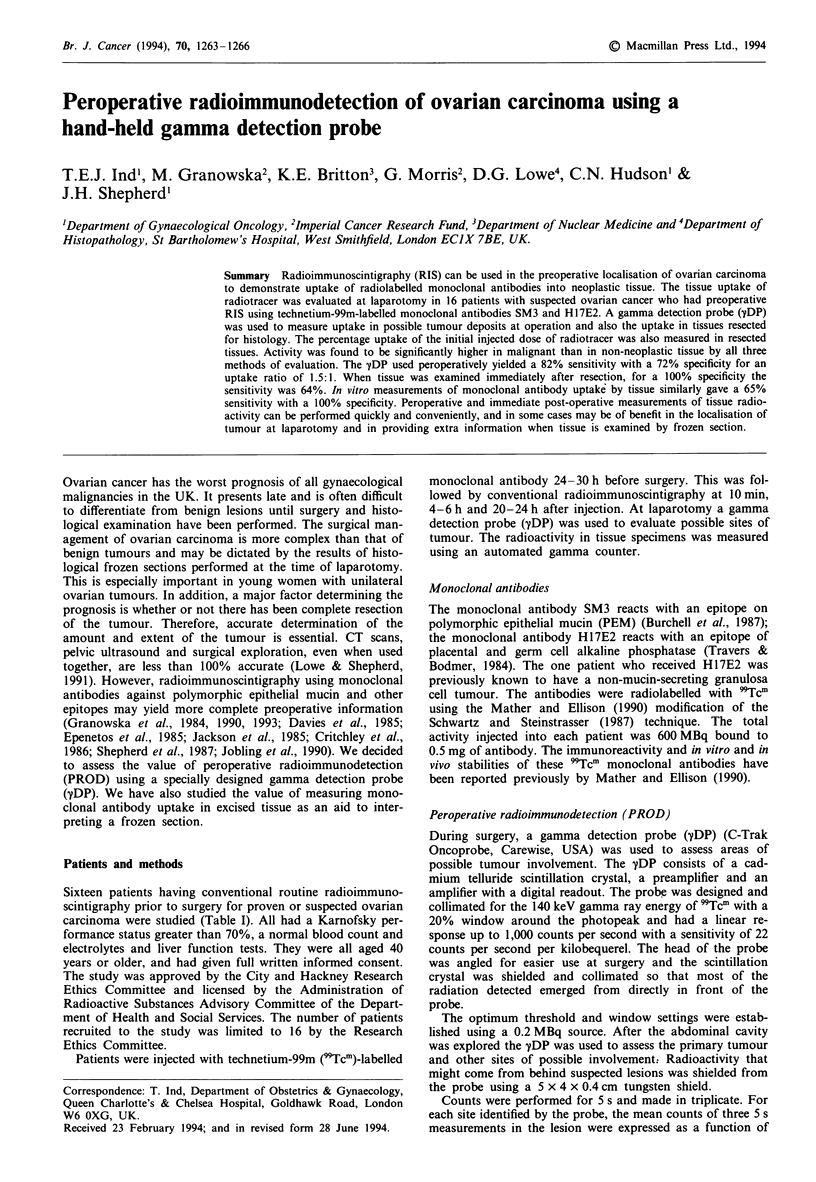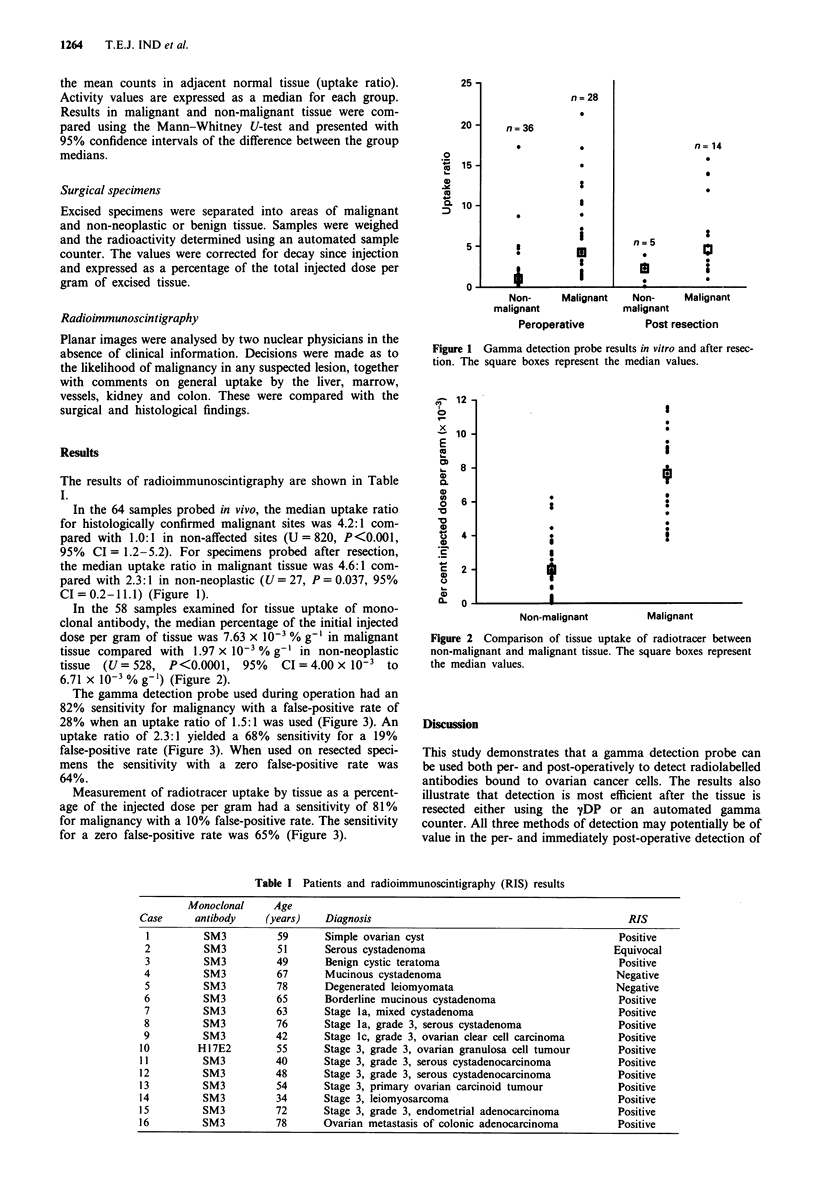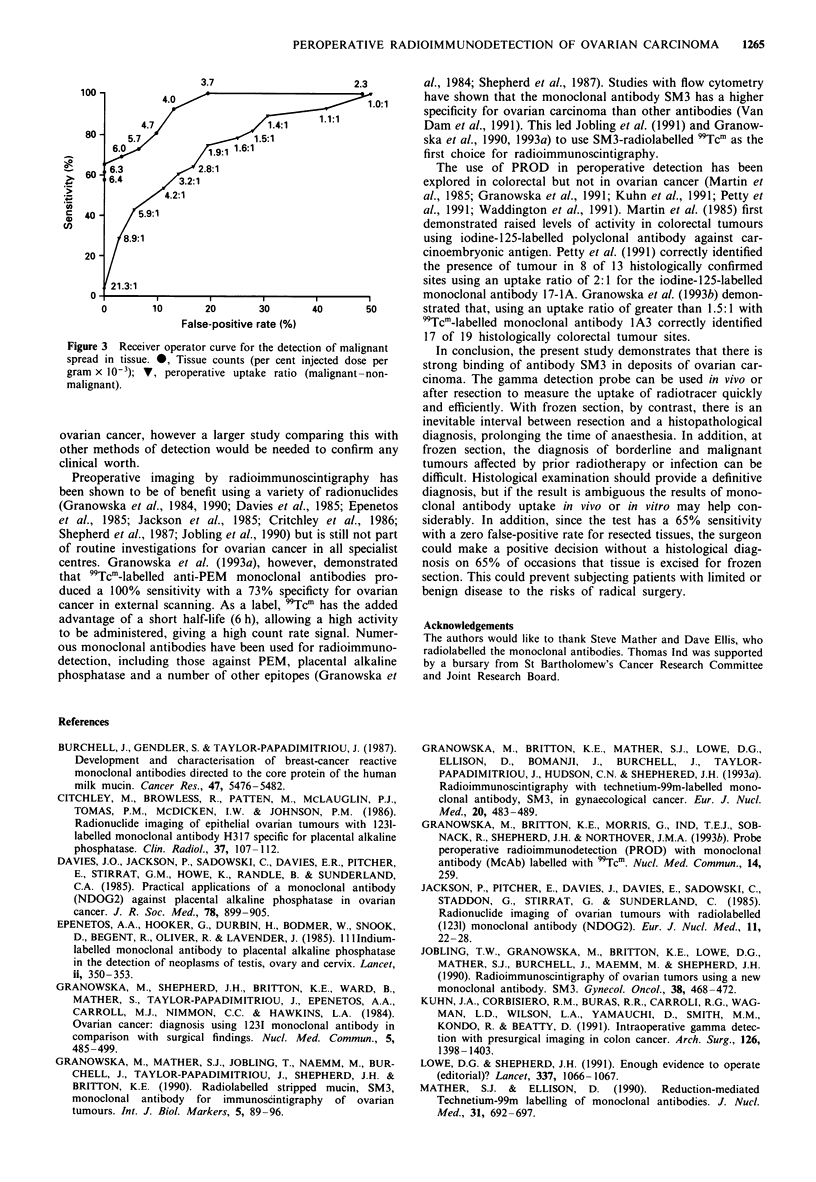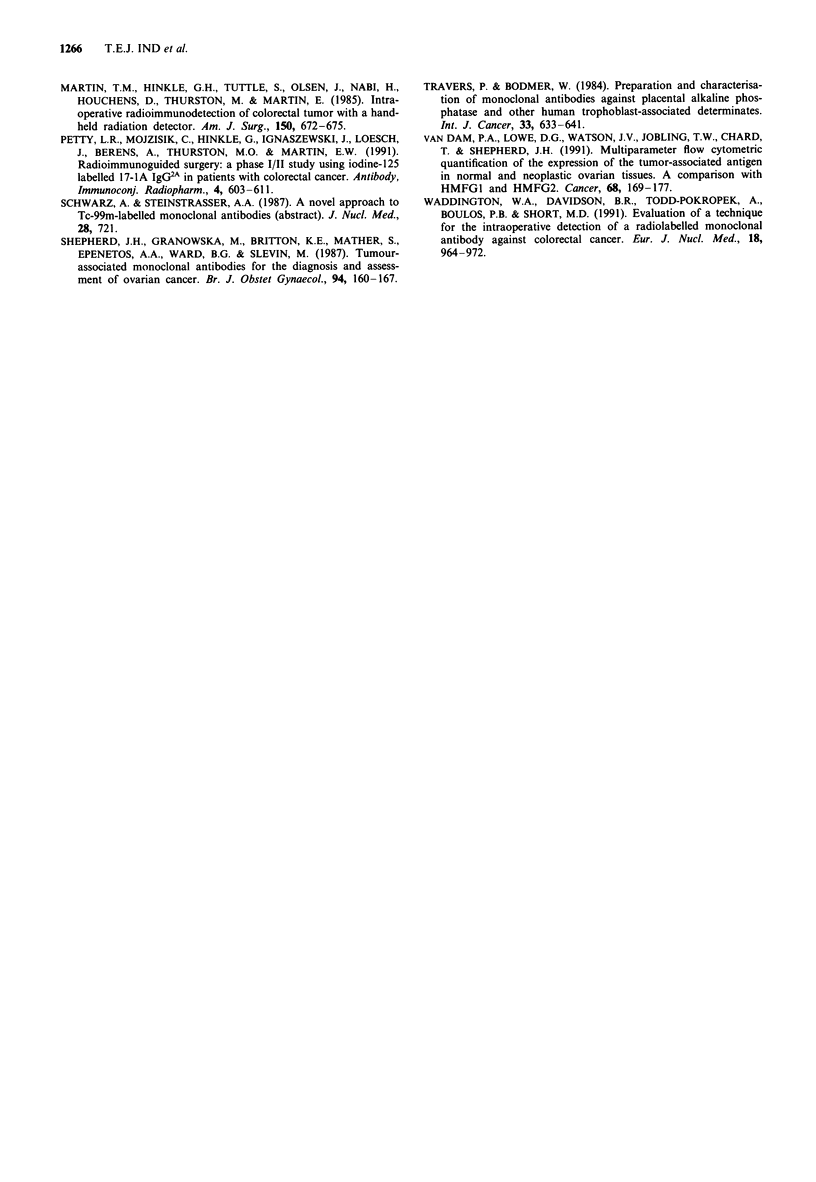# Peroperative radioimmunodetection of ovarian carcinoma using a hand-held gamma detection probe.

**DOI:** 10.1038/bjc.1994.484

**Published:** 1994-12

**Authors:** T. E. Ind, M. Granowska, K. E. Britton, G. Morris, D. G. Lowe, C. N. Hudson, J. H. Shepherd

**Affiliations:** Department of Gynaecological Oncology, St Bartholomew's Hospital, West Smithfield, London, UK.

## Abstract

Radioimmunoscintigraphy (RIS) can be used in the preoperative localisation of ovarian carcinoma to demonstrate uptake of radiolabelled monoclonal antibodies into neoplastic tissue. The tissue uptake of radiotracer was evaluated at laparotomy in 16 patients with suspected ovarian cancer who had preoperative RIS using technetium-99m-labelled monoclonal antibodies SM3 and H17E2. A gamma detection probe (gamma DP) was used to measure uptake in possible tumour deposits at operation and also the uptake in tissues resected for histology. The percentage uptake of the initial injected dose of radiotracer was also measured in resected tissues. Activity was found to be significantly higher in malignant than in non-neoplastic tissue by all three methods of evaluation. The gamma DP used peroperatively yielded a 82% sensitivity with a 72% specificity for an uptake ratio of 1.5:1. When tissue was examined immediately after resection, for a 100% specificity the sensitivity was 64%. In vitro measurements of monoclonal antibody uptake by tissue similarly gave a 65% sensitivity with a 100% specificity. Peroperative and immediate post-operative measurements of tissue radioactivity can be performed quickly and conveniently, and in some cases may be of benefit in the localisation of tumour at laparotomy and in providing extra information when tissue is examined by frozen section.


*Br. J. Cancer* (1994), **70**, 1263–1266 © Macmillan Press Ltd., 1994

### Peroperative radioimmunodetection of ovarian carcinoma using a hand-held gamma detection probe

T.E.J. Ind[1], M. Granowska[2], K.E. Britton[3], G. Morris[2], D.G. Lowe[4], C.N. Hudson[1] & J.H. Shepherd[1]

[1]*Department of Gynaecological Oncology,* [2]*Imperial Cancer Research Fund,* [3]*Department of Nuclear Medicine and* [4]*Department of Histopathology, St Bartholomew's Hospital, West Smithfield, London EC1X 7BE, UK.*

**Summary** Radioimmunoscintigraphy (RIS) can be used in the preoperative localisation of ovarian carcinoma to demonstrate uptake of radiolabelled monoclonal antibodies into neoplastic tissue. The tissue uptake of radiotracer was evaluated at laparotomy in 16 patients with suspected ovarian cancer who had preoperative RIS using technetium-99m-labelled monoclonal antibodies SM3 and H17E2. A gamma detection probe (γDP) was used to measure uptake in possible tumour deposits at operation and also the uptake in tissues resected for histology. The percentage uptake of the initial injected dose of radiotracer was also measured in resected tissues. Activity was found to be significantly higher in malignant than in non-neoplastic tissue by all three methods of evaluation. The γDP used peroperatively yielded a 82% sensitivity with a 72% specificity for an uptake ratio of 1.5:1. When tissue was examined immediately after resection, for a 100% specificity the sensitivity was 64%. *In vitro* measurements of monoclonal antibody uptake by tissue similarly gave a 65% sensitivity with a 100% specificity. Peroperative and immediate post-operative measurements of tissue radioactivity can be performed quickly and conveniently, and in some cases may be of benefit in the localisation of tumour at laparotomy and in providing extra information when tissue is examined by frozen section.

Ovarian cancer has the worst prognosis of all gynaecological malignancies in the UK. It presents late and is often difficult to differentiate from benign lesions until surgery and histological examination have been performed. The surgical management of ovarian carcinoma is more complex than that of benign tumours and may be dictated by the results of histological frozen sections performed at the time of laparotomy. This is especially important in young women with unilateral ovarian tumours. In addition, a major factor determining the prognosis is whether or not there has been complete resection of the tumour. Therefore, accurate determination of the amount and extent of the tumour is essential. CT scans, pelvic ultrasound and surgical exploration, even when used together, are less than 100% accurate (Lowe & Shepherd, 1991). However, radioimmunoscintigraphy using monoclonal antibodies against polymorphic epithelial mucin and other epitopes may yield more complete preoperative information (Granowska *et al.*, 1984, 1990, 1993; Davies *et al.*, 1985; Epenetos *et al.*, 1985; Jackson *et al.*, 1985; Critchley *et al.*, 1986; Shepherd *et al.*, 1987; Jobling *et al.*, 1990). We decided to assess the value of peroperative radioimmunodetection (PROD) using a specially designed gamma detection probe (γDP). We have also studied the value of measuring monoclonal antibody uptake in excised tissue as an aid to interpreting a frozen section.

### Patients and methods

Sixteen patients having conventional routine radioimmunoscintigraphy prior to surgery for proven or suspected ovarian carcinoma were studied (Table I). All had a Karnofsky performance status greater than 70%, a normal blood count and electrolytes and liver function tests. They were all aged 40 years or older, and had given full written informed consent. The study was approved by the City and Hackney Research Ethics Committee and licensed by the Administration of Radioactive Substances Advisory Committee of the Department of Health and Social Services. The number of patients recruited to the study was limited to 16 by the Research Ethics Committee.

Patients were injected with technetium-99m ($^{99}Tc^{m}$)-labelled monoclonal antibody 24–30 h before surgery. This was followed by conventional radioimmunoscintigraphy at 10 min, 4–6 h and 20–24 h after injection. At laparotomy a gamma detection probe (γDP) was used to evaluate possible sites of tumour. The radioactivity in tissue specimens was measured using an automated gamma counter.

*Monoclonal antibodies*

The monoclonal antibody SM3 reacts with an epitope on polymorphic epithelial mucin (PEM) (Burchell *et al.*, 1987); the monoclonal antibody H17E2 reacts with an epitope of placental and germ cell alkaline phosphatase (Travers & Bodmer, 1984). The one patient who received H17E2 was previously known to have a non-mucin-secreting granulosa cell tumour. The antibodies were radiolabelled with $^{99}Tc^{m}$ using the Mather and Ellison (1990) modification of the Schwartz and Steinstrasser (1987) technique. The total activity injected into each patient was 600 MBq bound to 0.5 mg of antibody. The immunoreactivity and *in vitro* and *in vivo* stabilities of these $^{99}Tc^{m}$ monoclonal antibodies have been reported previously by Mather and Ellison (1990).

*Peroperative radioimmunodetection (PROD)*

During surgery, a gamma detection probe (γDP) (C-Trak Oncoprobe, Carewise, USA) was used to assess areas of possible tumour involvement. The γDP consists of a cadmium telluride scintillation crystal, a preamplifier and an amplifier with a digital readout. The probe was designed and collimated for the 140 keV gamma ray energy of $^{99}Tc^{m}$ with a 20% window around the photopeak and had a linear response up to 1,000 counts per second with a sensitivity of 22 counts per second per kilobequerel. The head of the probe was angled for easier use at surgery and the scintillation crystal was shielded and collimated so that most of the radiation detected emerged from directly in front of the probe.

The optimum threshold and window settings were established using a 0.2 MBq source. After the abdominal cavity was explored the γDP was used to assess the primary tumour and other sites of possible involvement. Radioactivity that might come from behind suspected lesions was shielded from the probe using a 5 × 4 × 0.4 cm tungsten shield.

Counts were performed for 5 s and made in triplicate. For each site identified by the probe, the mean counts of three 5 s measurements in the lesion were expressed as a function of

Correspondence: T. Ind, Department of Obstetrics & Gynaecology, Queen Charlotte's & Chelsea Hospital, Goldhawk Road, London W6 0XG, UK.
Received 23 February 1994; and in revised form 28 June 1994.

the mean counts in adjacent normal tissue (uptake ratio). Activity values are expressed as a median for each group. Results in malignant and non-malignant tissue were compared using the Mann–Whitney U-test and presented with 95% confidence intervals of the difference between the group medians.

### *Surgical specimens*

Excised specimens were separated into areas of malignant and non-neoplastic or benign tissue. Samples were weighed and the radioactivity determined using an automated sample counter. The values were corrected for decay since injection and expressed as a percentage of the total injected dose per gram of excised tissue.

### *Radioimmunoscintigraphy*

Planar images were analysed by two nuclear physicians in the absence of clinical information. Decisions were made as to the likelihood of malignancy in any suspected lesion, together with comments on general uptake by the liver, marrow, vessels, kidney and colon. These were compared with the surgical and histological findings.

### Results

The results of radioimmunoscintigraphy are shown in Table I.

In the 64 samples probed *in vivo*, the median uptake ratio for histologically confirmed malignant sites was 4.2:1 compared with 1.0:1 in non-affected sites ($U = 820$, $P<0.001$, 95% CI = 1.2–5.2). For specimens probed after resection, the median uptake ratio in malignant tissue was 4.6:1 compared with 2.3:1 in non-neoplastic ($U = 27$, $P = 0.037$, 95% CI = 0.2–11.1) (Figure 1).

In the 58 samples examined for tissue uptake of monoclonal antibody, the median percentage of the initial injected dose per gram of tissue was $7.63 \times 10^{-3}$ % $g^{-1}$ in malignant tissue compared with $1.97 \times 10^{-3}$ % $g^{-1}$ in non-neoplastic tissue ($U = 528$, $P<0.0001$, 95% CI = $4.00 \times 10^{-3}$ to $6.71 \times 10^{-3}$ % $g^{-1}$) (Figure 2).

The gamma detection probe used during operation had an 82% sensitivity for malignancy with a false-positive rate of 28% when an uptake ratio of 1.5:1 was used (Figure 3). An uptake ratio of 2.3:1 yielded a 68% sensitivity for a 19% false-positive rate (Figure 3). When used on resected specimens the sensitivity with a zero false-positive rate was 64%.

Measurement of radiotracer uptake by tissue as a percentage of the injected dose per gram had a sensitivity of 81% for malignancy with a 10% false-positive rate. The sensitivity for a zero false-positive rate was 65% (Figure 3).

**Figure 1** Gamma detection probe results *in vitro* and after resection. The square boxes represent the median values.

**Figure 2** Comparison of tissue uptake of radiotracer between non-malignant and malignant tissue. The square boxes represent the median values.

### Discussion

This study demonstrates that a gamma detection probe can be used both per- and post-operatively to detect radiolabelled antibodies bound to ovarian cancer cells. The results also illustrate that detection is most efficient after the tissue is resected either using the γDP or an automated gamma counter. All three methods of detection may potentially be of value in the per- and immediately post-operative detection of

**Table I** Patients and radioimmunoscintigraphy (RIS) results

| *Case* | *Monoclonal antibody* | *Age (years)* | *Diagnosis* | *RIS* |
|---|---|---|---|---|
| 1 | SM3 | 59 | Simple ovarian cyst | Positive |
| 2 | SM3 | 51 | Serous cystadenoma | Equivocal |
| 3 | SM3 | 49 | Benign cystic teratoma | Positive |
| 4 | SM3 | 67 | Mucinous cystadenoma | Negative |
| 5 | SM3 | 78 | Degenerated leiomyomata | Negative |
| 6 | SM3 | 65 | Borderline mucinous cystadenoma | Positive |
| 7 | SM3 | 63 | Stage 1a, mixed cystadenoma | Positive |
| 8 | SM3 | 76 | Stage 1a, grade 3, serous cystadenoma | Positive |
| 9 | SM3 | 42 | Stage 1c, grade 3, ovarian clear cell carcinoma | Positive |
| 10 | H17E2 | 55 | Stage 3, grade 3, ovarian granulosa cell tumour | Positive |
| 11 | SM3 | 40 | Stage 3, grade 3, serous cystadenocarcinoma | Positive |
| 12 | SM3 | 48 | Stage 3, grade 3, serous cystadenocarcinoma | Positive |
| 13 | SM3 | 54 | Stage 3, primary ovarian carcinoid tumour | Positive |
| 14 | SM3 | 34 | Stage 3, leiomyosarcoma | Positive |
| 15 | SM3 | 72 | Stage 3, grade 3, endometrial adenocarcinoma | Positive |
| 16 | SM3 | 78 | Ovarian metastasis of colonic adenocarcinoma | Positive |

**Figure 3** Receiver operator curve for the detection of malignant spread in tissue. ●, Tissue counts (per cent injected dose per gram × $10^{-3}$); ▼, peroperative uptake ratio (malignant–non-malignant).

ovarian cancer, however a larger study comparing this with other methods of detection would be needed to confirm any clinical worth.

Preoperative imaging by radioimmunoscintigraphy has been shown to be of benefit using a variety of radionuclides (Granowska *et al.*, 1984, 1990; Davies *et al.*, 1985; Epenetos *et al.*, 1985; Jackson *et al.*, 1985; Critchley *et al.*, 1986; Shepherd *et al.*, 1987; Jobling *et al.*, 1990) but is still not part of routine investigations for ovarian cancer in all specialist centres. Granowska *et al.* (1993*a*), however, demonstrated that $^{99}Tc^{m}$-labelled anti-PEM monoclonal antibodies produced a 100% sensitivity with a 73% specificty for ovarian cancer in external scanning. As a label, $^{99}Tc^{m}$ has the added advantage of a short half-life (6 h), allowing a high activity to be administered, giving a high count rate signal. Numerous monoclonal antibodies have been used for radioimmunodetection, including those against PEM, placental alkaline phosphatase and a number of other epitopes (Granowska *et al.*, 1984; Shepherd *et al.*, 1987). Studies with flow cytometry have shown that the monoclonal antibody SM3 has a higher specificity for ovarian carcinoma than other antibodies (Van Dam *et al.*, 1991). This led Jobling *et al.* (1991) and Granowska *et al.*, 1990, 1993*a*) to use SM3-radiolabelled $^{99}Tc^{m}$ as the first choice for radioimmunoscintigraphy.

The use of PROD in peroperative detection has been explored in colorectal but not in ovarian cancer (Martin *et al.*, 1985; Granowska *et al.*, 1991; Kuhn *et al.*, 1991; Petty *et al.*, 1991; Waddington *et al.*, 1991). Martin *et al.* (1985) first demonstrated raised levels of activity in colorectal tumours using iodine-125-labelled polyclonal antibody against carcinoembryonic antigen. Petty *et al.* (1991) correctly identified the presence of tumour in 8 of 13 histologically confirmed sites using an uptake ratio of 2:1 for the iodine-125-labelled monoclonal antibody 17-1A. Granowska *et al.* (1993*b*) demonstrated that, using an uptake ratio of greater than 1.5:1 with $^{99}Tc^{m}$-labelled monoclonal antibody 1A3 correctly identified 17 of 19 histologically colorectal tumour sites.

In conclusion, the present study demonstrates that there is strong binding of antibody SM3 in deposits of ovarian carcinoma. The gamma detection probe can be used *in vivo* or after resection to measure the uptake of radiotracer quickly and efficiently. With frozen section, by contrast, there is an inevitable interval between resection and a histopathological diagnosis, prolonging the time of anaesthesia. In addition, at frozen section, the diagnosis of borderline and malignant tumours affected by prior radiotherapy or infection can be difficult. Histological examination should provide a definitive diagnosis, but if the result is ambiguous the results of monoclonal antibody uptake *in vivo* or *in vitro* may help considerably. In addition, since the test has a 65% sensitivity with a zero false-positive rate for resected tissues, the surgeon could make a positive decision without a histological diagnosis on 65% of occasions that tissue is excised for frozen section. This could prevent subjecting patients with limited or benign disease to the risks of radical surgery.

**Acknowledgements**

The authors would like to thank Steve Mather and Dave Ellis, who radiolabelled the monoclonal antibodies. Thomas Ind was supported by a bursary from St Bartholomew's Cancer Research Committee and Joint Research Board.

### References

BURCHELL, J., GENDLER, S. & TAYLOR-PAPADIMITRIOU, J. (1987). Development and characterisation of breast-cancer reactive monoclonal antibodies directed to the core protein of the human milk mucin. *Cancer Res.*, **47**, 5476–5482.

CITCHLEY, M., BROWLESS, R., PATTEN, M., MCLAUGLIN, P.J., TOMAS, P.M., MCDICKEN, I.W. & JOHNSON, P.M. (1986). Radionuclide imaging of epithelial ovarian tumours with 123I-labelled monoclonal antibody H317 specific for placental alkaline phosphatase. *Clin. Radiol.*, **37**, 107–112.

DAVIES, J.O., JACKSON, P., SADOWSKI, C., DAVIES, E.R., PITCHER, E., STIRRAT, G.M., HOWE, K., RANDLE, B. & SUNDERLAND, C.A. (1985). Practical applications of a monoclonal antibody (NDOG2) against placental alkaline phosphatase in ovarian cancer. *J. R. Soc. Med.*, **78**, 899–905.

EPENETOS, A.A., HOOKER, G., DURBIN, H., BODMER, W., SNOOK, D., BEGENT, R., OLIVER, R. & LAVENDER, J. (1985). 111Indium-labelled monoclonal antibody to placental alkaline phosphatase in the detection of neoplasms of testis, ovary and cervix. *Lancet*, **ii**, 350–353.

GRANOWSKA, M., SHEPHERD, J.H., BRITTON, K.E., WARD, B., MATHER, S., TAYLOR-PAPADIMITRIOU, J., EPENETOS, A.A., CARROLL, M.J., NIMMON, C.C. & HAWKINS, L.A. (1984). Ovarian cancer: diagnosis using 123I monoclonal antibody in comparison with surgical findings. *Nucl. Med. Commun.*, **5**, 485–499.

GRANOWSKA, M., MATHER, S.J., JOBLING, T., NAEMM, M., BURCHELL, J., TAYLOR-PAPADIMITRIOU, J., SHEPHERD, J.H. & BRITTON, K.E. (1990). Radiolabelled stripped mucin, SM3, monoclonal antibody for immunoscintigraphy of ovarian tumours. *Int. J. Biol. Markers*, **5**, 89–96.

GRANOWSKA, M., BRITTON, K.E., MATHER, S.J., LOWE, D.G., ELLISON, D., BOMANJI, J., BURCHELL, J., TAYLOR-PAPADIMITRIOU, J., HUDSON, C.N. & SHEPHERED, J.H. (1993*a*). Radioimmunoscintigraphy with technetium-99m-labelled monoclonal antibody, SM3, in gynaecological cancer. *Eur. J. Nucl. Med.*, **20**, 483–489.

GRANOWSKA, M., BRITTON, K.E., MORRIS, G., IND, T.E.J., SOBNACK, R., SHEPHERD, J.H. & NORTHOVER, J.M.A. (1993*b*). Probe peroperative radioimmunodetection (PROD) with monoclonal antibody (McAb) labelled with $^{99}Tc^{m}$. *Nucl. Med. Commun.*, **14**, 259.

JACKSON, P., PITCHER, E., DAVIES, J., DAVIES, E., SADOWSKI, C., STADDON, G., STIRRAT, G. & SUNDERLAND, C. (1985). Radionuclide imaging of ovarian tumours with radiolabelled (123I) monoclonal antibody (NDOG2). *Eur. J. Nucl. Med.*, **11**, 22–28.

JOBLING, T.W., GRANOWSKA, M., BRITTON, K.E., LOWE, D.G., MATHER, S.J., BURCHELL, J., MAEMM, M. & SHEPHERD, J.H. (1990). Radioimmunoscintigraphy of ovarian tumors using a new monoclonal antibody. SM3. *Gynecol. Oncol.*, **38**, 468–472.

KUHN, J.A., CORBISIERO, R.M., BURAS, R.R., CARROLI, R.G., WAGMAN, L.D., WILSON, L.A., YAMAUCHI, D., SMITH, M.M., KONDO, R. & BEATTY, D. (1991). Intraoperative gamma detection with presurgical imaging in colon cancer. *Arch. Surg.*, **126**, 1398–1403.

LOWE, D.G. & SHEPHERD, J.H. (1991). Enough evidence to operate (editorial)? *Lancet*, **337**, 1066–1067.

MATHER, S.J. & ELLISON, D. (1990). Reduction-mediated Technetium-99m labelling of monoclonal antibodies. *J. Nucl. Med.*, **31**, 692–697.

MARTIN, T.M., HINKLE, G.H., TUTTLE, S., OLSEN, J., NABI, H., HOUCHENS, D., THURSTON, M. & MARTIN, E. (1985). Intraoperative radioimmunodetection of colorectal tumor with a hand-held radiation detector. *Am. J. Surg.*, **150**, 672-675.

PETTY, L.R., MOJZISIK, C., HINKLE, G., IGNASZEWSKI, J., LOESCH, J., BERENS, A., THURSTON, M.O. & MARTIN, E.W. (1991). Radioimmunoguided surgery: a phase I/II study using iodine-125 labelled 17-1A $IgG^{2A}$ in patients with colorectal cancer. *Antibody, Immunoconj. Radiopharm.*, **4**, 603-611.

SCHWARZ, A. & STEINSTRASSER, A.A. (1987). A novel approach to Tc-99m-labelled monoclonal antibodies (abstract). *J. Nucl. Med.*, **28**, 721.

SHEPHERD, J.H., GRANOWSKA, M., BRITTON, K.E., MATHER, S., EPENETOS, A.A., WARD, B.G. & SLEVIN, M. (1987). Tumour-associated monoclonal antibodies for the diagnosis and assessment of ovarian cancer. *Br. J. Obstet Gynaecol.*, **94**, 160-167.

TRAVERS, P. & BODMER, W. (1984). Preparation and characterisation of monoclonal antibodies against placental alkaline phosphatase and other human trophoblast-associated determinates. *Int. J. Cancer*, **33**, 633-641.

VAN DAM, P.A., LOWE, D.G., WATSON, J.V., JOBLING, T.W., CHARD, T. & SHEPHERD, J.H. (1991). Multiparameter flow cytometric quantification of the expression of the tumor-associated antigen in normal and neoplastic ovarian tissues. A comparison with HMFG1 and HMFG2. *Cancer*, **68**, 169-177.

WADDINGTON, W.A., DAVIDSON, B.R., TODD-POKROPEK, A., BOULOS, P.B. & SHORT, M.D. (1991). Evaluation of a technique for the intraoperative detection of a radiolabelled monoclonal antibody against colorectal cancer. *Eur. J. Nucl. Med.*, **18**, 964-972.